# 

**Published:** 1896-04-04

**Authors:** 


### The Hospital

AN INSTITUTIONAL JOURNAL OF

THE MEDICAL SCIENCES AND HOSPITAL ADMINISTRATION,

INCLUDING

### A SPECIAL SUPPLEMENT FOR NURSES.

EDITED BY

HENRY C. BURDETT.

IN TWO VOLUMES ANNUALLY.

158763.
LIBRARY

**VOLUME XX.**

April, 1896, to September, 1896.

London:

PUBLISHED BY THE SCIENTIFIC PRESS (LIMITED), AT THEIR OFFICE, 428, STRAND, W.C.

MDCCCXCVI.

WI
H069X

### INDEX TO VOL. XX., 1896.

*** Articles under the general heading *Medical Progress and Hospital Clinics* are distinguished by the initial (C.) for *Hospital Clinics*, and by (P.) for the rest of the series. (W.) distinguishes the *Institutional Workshop* articles, and (A.H.) the notes under the general title *Around the Hospitals*.

Abdomen, Diseases of the (P.), 191
Abdominal Injuries Without External Wounds (P.), 264
Abdominal Section, Shock after (P.), 263
Abdominal Sepsis Treated by Intra-venous Serum (P.), 264
Aberdeen Royal Asylum New Hospital (W.), 382
Abscess of the Lung (P.), 126
Abscess, Retropharyngeal (P.), 362
Abscess, Sub-diaphragmatic (P.), 125
Abscess, Subperiosteal Mastoid (P.), 161
Accrington Cottage Hospital (W.), 379
Acetonuria and Coma (P.), 92
Acne (P.), 310
Actinomycosis (P.), 397
Actinomycosis and Anthrax (P.), 109
Alcohol, Effects of (P.), 409
Alcoholic Drinks (P.), 25
Alcoholic Stimulation (C.), 292. *and see p.* 349
Alcoholism, a Fruit Cure for, 119
Aldershot, New Hospital at (W.), 365
Amusements of Poor Women, The, 137
Anacrotic Pulse (P.), 280
Anæmia, New Methods of Treating (P.), 345
Anæmia (Pernicious) and Chlorosis (P.), 241
Anæsthesia, General, A Preliminary Precaution (P.), 128
Anastomosis, Intestinal (P.), 9, 10, 359
Anglo-German Hospital, Rosario (W.), 331
Anthrax and Actinomycosis (P.), 109
Antiseptics (P.), 41, 411
Anti-toxin Diphtheria, *see p.* 44
Anti-toxin, The Report on Diphtheria, 18
Anti-vivisection Literature, 171
Antral Sinusitis and Empyema (P.), 111
Appendicitis (P.), 345
Appendicitis, The Pathology of (C.), 124, 141
Around the Hospitals (A.H.), 180, 232, 432
Arterio-Sclerosis (P.), 277
Arthritis, Rheumatoid (P.), 158
Arthrodesis (P.), 282
Asthma (C.), 175, 206
Battle of the Clubs, The, 236
Bearsden Convalescent Home (W.), 82
Bed, Invalid. *See p.* 46
Bicycle Saddles, Pressure of, on the Perineum (P.), 226
Birmingham Hospital Saturday Fund, 433
Bladder, Tuberculous Disease of the (P.), 427
Bladder, Tumours and Pouches of the (P.), 208
Blood, Diseases of the (P.), 241
Boat Race, Mr. G. Bourne on the Cost of the, 20
Boat Race, The Physiological Cost of the, 2, 28
Bone Grafting for Cranial Defect (P.), 144
Book World of Medicine, The (*Books, Magazines, and Pamphlets reviewed or noticed and articles relating to*):—
Allingham. Diagnosis of Diseases of the Rectum, 233
Allingham. Diseases of the Rectum, Anus, &c., 99
American Medico-Psychological Association, Proceedings, 333
Barclay. Southall's Materia Medica, 269
Bell. Sterility, 383
Bergey. *See Billings*
Billings, Weir-Mitchell, and Bergey. The Composition of Expired Air, 99
Brothers. Infantile Mortality During Childbirth, 383
Burdett, Hospitals and Charities, 1896, 215
Byford. Manual of Gynæcology, 83
Chemists and Druggists, Directory of, 199
City of London Directory, 133
Clarkson. A Text Book of Histology, 401
Daniell. Physics for Students of Medicine, 285
Dentists' Register, 1896, 65
Fox. Strathpeffer Spa, 333
Goldsbrough. Some Prolegomena to a Philosophy of Medicine, 167, 214
Heider. *See Korschelt*
Hertwig. The Biological Problem of the Day, 333
Horent. A New Treatment of So-called Incurably Deaf People, 285
Hutton. The Vaccination Question, 15
Korschelt and Heider (trans. by Mark and Woodworth). Comparative Embryology, 65
Lewis. Early Scoliosis or Curable Curvature of the Spine, 15
McKendrick. Elementary Human Physiology, 251
Mark. *See Korschelt*
Medical Register, 1896, 65
Mitchell's Newspaper Press Directory, 1896, 133
Morris. Injuries and Diseases of the Genital and Urinary Organs, 47
Book World of Medicine (*continued*)
Premature Burial, 47
Richardson. Biological Experimentation, 233
Robertson. Clinical Diagnosis, 199
Sanders. An Encyclopædia of Gardening, 15
Scoville. The Art of Compounding, 47
Southall. *See Barclay*
Startin. A Pharmacopœia for Diseases of the Skin, 15
Stevenson. Uninhabitable and Insanitary Houses, 133
Street's List of Newspapers, 199
Sutton. Surgical Diseases of the Ovaries, &c., 269
Thoma. Text-book of General Pathology, &c., 83
Thorne. The Schott Treatment of Chronic Disease of the Heart, 65
Tyson. Guide to the Examination, 149
Van Horlingen. Handbook of the Diagnosis and Treatment of Skin Diseases, 47
Weber and Webber. Spas and Mineral Waters of Europe, 251
Weir-Mitchell. *See Billings*
Willing's British and Irish Press Guide, 133
Woodworth. *See Korschelt*
Year Book of the Scientific and Learned Societies, 233
Yorke-Davies. Health and Condition in the Active and the Sedentary, 251
"Box Begging," 171
Brain Lesions, Optic Neuritis in (P.), 143
Brain Surgery (P.), 109, 143, 160
Brain Tumours (P.), 109
Breaking-strain, The (C.), 122
Bright's Disease, Early Diagnosis of, 202
British Medical Association and Medical Defence, 272
British Medical Association and their Midwives' Bill, 218
Bronchial Tubes, Foreign Bodies in the (P.), 125
Bronchitis, Notes on the Treatment of (C.), 140, 174, 190
Burial of the Dead, The, 51
Burns, Picric Acid in the Treatment of (C.), 78, 107
Camden, N.S.W., Carrington Convalescent Hospital (A.H.), 432
Cancer (P.), 426
Cancer of the Rectum (C.), 356
Cancer of the Tongue (C.), 306
Capital Punishment, 189
Carbohydrate Food (P.), 411
Carbohydrate Starvation in Typhoid Fever, 3, *and see p.* 53
Carcinoma of the Rectum (P.), 311
Catheterism, Posterior (P.), 225
Central Hospital Board for London, A (W.), 45
Central Hospitals Board, The (W.), 97, 113, 129, 147
Central Hospitals Board Question, The, 67
Cephalhydrocoele, Traumatic (P.), 144
Cerebellar Lesions, A New Symptom Localising (P.), 143
Cerebral Concussion (P.), 144
Cerebral Sinuses, Thrombosis of (P.), 160
Ceruminous Accumulations, The Removal of (P.), 245
Cervical Rib, A (P.), 126
Cervix Uteri, Epithelioma of (P.), 194
Charity, Capacity, Continuity, 169
Chelsea Hospital for Women, Cat's Paws at the, 153
Chicken Pox in an Adult Pregnant Woman (C.), 73
Children, Diseases of (C.), 376
Children, Diseases of (P.), 8, 360
Children, The Medical Care of, in Durham, 186
Cholalogues (P.), 327
Cholecystomy for Lithiasis (P.), 377
Choledochotomy for Calculi (P.), 377
Cholera (P.), 108, 357
Cholera Controversy, A Half-century (C), 72
Cholera in Egypt, 237
Chronic Varicose Ulcers of the Leg, The Treatment of (C.), 424
Circulatory System, Diseases of the (P.), 125, 142, 260, 277
Clarke's Bobbin (Intestinal Surgery), 359
Classification in Workhouses, 355, 370
Clinic, A Dispensary (C.), 356
Clinical Recollections (C.), 106
Clinical Society, The, 5
Clitoris, Primary Malignant Disease of the (P.), 194
Club-foot (P.), 282
Club-foot (Congenital), The Treatment of in Infancy (C), 408
Cochlear Apoplexy (P.), 227
Colectomy (P.), 11
Colliery Explosions, The Prevention of, 288
Colotomy (P.), 11
Coma (Diabetes) (P.), 92
Commissariat, The, Hospital Expenditure (W.), 61, 79, 163, 247, 265, 283, 318, 329, 347, 363, 399, 413, 428
Compulsory Revaccination, 335
Condensed Foods (P.), 26
Construction Notes (W.), 30, 46, 63, 82, 165, 183, 231, 250, 284, 332, 365, 382
Consumption: Is Consumption on the Decrease? 303
Convalescent Home at Llanfairfechan (W.), 63
Coroners' Juries and Hospitals, 337
Coronilla (P.), 278
Correspondence. *See Editor's Letter-Box*
Cottage Hospitals (W.), 30, 63, 213, 231, 267, 379
Coxa Vara (P.), 281
Cranial Defect, Bone Grafting for (P.), 144
Craniectomy for Microcephalus (P.), 143
Cremation, The Progress of, in America, 121
Cretinism, Sporadic Value of Thyroid Treatment in (C.), 376
Criminal Mania, 19, 37, 53, 71
"Cure," A New, for Drunkenness, 289
Cycling and Heart Disease, 203
Cyst, Pancreatic (P.), 26
Cysts, Mesenteric (P.), 360
Death, Causes of. *See p.* 122
Death Rates and Salubrity, 421
Deciduoma Malignum (P.), 178
Deformities, Postural, of the Trunk (P.), 280
Deformities, Rickety, of the Long Bones (P.), 282
Dentistry, The Advance of, 337
Derbyshire Hospital for Sick Children (W.), 30
Derbyshire Royal Infirmary (W.), 29
Dermatology, Progress in (P.), 307, 326
Diabetes (P.), 57, 74, 91
Diabetes, Bronzed (P.), 91
Diabetic Treatment, Ferments in (P.), 161
Diarrhœa, Antiseptics for Infant (P.), 210
Diet for School Boys, 275, 291
Digitalin Crystallised (P.), 278
Diphtheria Anti-toxin, 18, 44
Diphtheria, Etiology of (C.), 258
Diphtheria in Soil, in Cows, and in Human Beings, 353
Disinfection by the Vapour of Formaldehyde (C.), 276
Dispense? Should English Doctors, 151
Doctors: Have Doctors Rights? 369
Doctors and Midwives 336
Drink Mortality at Liverpool, 353
Dropsy (P.), 260
Drug Rashes (P.), 326
Drumhead, Rupture of the, by Blows upon the Ear (P.), 226
Drunkards, The Treatment of Criminal, 405
Dundee Infirmary Nurses' Home (W.), 82
Ear. *See Middle Ear*, Otology
Ectopic Gestation (P.), 179 (C.), 409
Eczema (P.), 307
Editor's Letter Box, 44, 115, 132, 180, 214, 267, 349
Education, Some Medical Aspects of, 119
Election of Direct Representatives, The, 287
Empyema (P.), 111
Endocarditis, Acute (P.), 278
Endocarditis, Infective, in Tonsilitis (P.), 278
Endocarditis, Tuberculous (P.), 278
Epilepsy, Traumatic (P.), 144
Ethmoid, Fracture of, with Basal Meningitis (P.), 145
Ethmoiditis, Suppurating (P.), 94
Eucaine, A New Substitute for Cocaine (P.), 328
Examination of Medical Students, The, 185
Examiner, The Decline and Fall of the, 369
Exostosis of the External Meatus (P.), 228
Extravasation of Urine (P.), 225
Eyesight of School Children, 271
Facial Paralysis. *See p.* 244
Femoral Hernia, The Treatment of (C.), 23
Fever, Scarlet (P.), 108
Fevers, Tropical (P.), 107
Fistula in Ano (P.), 310
Flat-foot (P.), 282
Flat-foot, Acquired, The Treatment of (C.), 7
Fleetwood Cottage Hospital (W.), 63
Foods and Digestion (P.), 25
Formaldehyde, Disinfection by (C.), 276, 322
Formalin (P.), 296
Formalin Gelatine, 428
Fractures and Dislocations (P.), 42, 60
Fractures and Dislocations of Lower Extremity (P.), 43, 60
Fractures and Dislocations of Upper Extremity (P.), 60

"Free from Infection," 237
Friendly Societies and Revaccination, 103
Gall-bladder, Cancer of the (P.), 410
Gastric Ulcer, Perforating (P.), 325
Gastro-enterostomy (P.), 325
Gastrostomy (P.), 325
Genito-Urinary Surgery (P.), 177, 208, 426
Genu Recurvatum (P.), 281
Gestation Ectopic (P.), 179 (C.), 409
Glasgow Samaritan Hospital for Women (W.), 130
Glenard's Disease, Treatment of (P.), 263
Gout and Rheumatism (P.), 158
Grindings of Civilisation's Mill, The, 137
Groin, Tumours of the (C.) 223, 240
Guy's Hospital (W.), 195
Gynæcology, Progress in (P.), 193
Hæmorrhoids (P.), 312
Hair, Diseases of the (P.), 310
Hallux Valgus (P.), 282
Head Injury with Aphasia (P.), 144
Head Injuries (P.), 144
Heart Disease. *See Nauheim Treatment*
Heatherdene Home, Extension of (W.), 231
Hebrew Influence, 257
Helsingfors Surgical Hospital (W.), 414
Hepatic Abscess (P.), 377
Heredity, Modes of, 171
Hermite System at Netley, 275
Hernia, Femoral, The Treatment of (C.), 23
Hip-joint, Congenital Displacement of the (P.), 280
Holidays and Heredity, 237
Home Industry and the Sweating System, 205
Homes for the Dying (W.), 266, 431
Homœopathic Hospital, Melbourne (W.), 364
Hospital Administration (W.), 62, 230
Hospital Construction (W.), 131, 213, 249, 331, 364, 400, 430
Hospital Expenditure (W.), 61, 79, 163, 211, 229, 247, 265, 283, 313, 329, 347, 363, 399, 413, 429
Hospital Festivals and Meetings (W.), 13, 31, 64, 98, 114, 131, 148, 165, 183, 218, 267, 298, 315, 381
Hospital Mortuaries, 273
Hospital Saturday Fund in Danger, A, 403
ospital Ships (W.), 432
ospital Sunday Fund, Metropolitan, 181, 297, 299
OSPITAL SUNDAY SUPPLEMENT, SPECIAL:—
*Death the Destroyer*, 9
*Diseases from which Londoners Suffer*, 11
*Our Crowded London and its Hospitals*, 12
*Embarrassing Popularity*, 12
*Sanctuary of the Sick, The*, 13
*Rates, Charity, and Common Sense*, 14
*Word to Living Londoners*, 15
*Metropolitan Hospital Sunday Fund*, 16
*Hospital Sunday in London*, 17
*Work done by the Hospitals and Medical Charities in 1895*, 17
*Summary of the Work done in 1895*, 20
*Need and Value of Personal Devotion*, 20
*Hint to the Wise*, 20
pital, The Evolution of the Modern, 305, 321, 339
PITAL THE, The place of, in Medical Journalism, 17
ospitals, A New Danger for, 51
Hospitals Association, The, 80
Hospitals, Foreign (W.), 441
Hospitals in India (W.), 248
Hot Air as a Therapeutic Agent (P.), 295
Hotch-potch Reformers (W.), 62
House Drains and Disease at Bow, 203
Hydatid Cyst of the Liver (P.), 377
Hydrocephalus, Surgical Treatment of (P.), 144
Hydronephrosis—Its Causes, Diagnosis, and Treatment (C.), 341
Hydrophobia (P.), 109
Hyperpyrexia, Treatment of, by Apolysin (P.), 361
Hypertrophy (Glandular) in Aural Disease (P.), 243
Hypertrophy, Prostatic Treatment of (P.), 426
Ice Creams in Society and in the Streets, 137
Ignorant Editors and Struggling Hospitals, 219
India, Hospitals in (W.), 248
Indigestion, Infantile, 319
Infectious Diseases (P.), 357, 396
Infectious Fevers, Serum Injection in (P.), 162
Infective Diseases (P.), 58, 107
Inquests, Unnecessary, 153
Insane: The Story of the Insane from Year to Year, 132, 164, 212, 230, 314, 331, 348, 381, 416
Insanity, Suicidal Impulses in (C.), 90
Insanity, Traumatic (P.), 145
Intestinal Anastomosis, Maunsell's Method of (P.), 10
Intestinal Anastomosis, New Bobbins for (P.), 9
Intestinal Obstruction (P.), 11
Intestinal Resection (P.), 9, 359
Intestine, Circular Suture of the (P.), 9
Intestine, Simple Stricture of the (P.), 11
Intestine, Wounds of the (P.), 358
Intestines (P.), 192
Intestines, Surgery of the (P.), 9, 358
Intussusception (P.), 11
Iodates, The Therapeutic Value of (P.), 12
Jaundice, Hœmatagenous (P.), 410
Jenner, A Hundred Years After, 102
Jenner Society, The. *See p.* 132
Jewish Babies, The Life and Death of, 4
Johnson. Pathology of the Contracted Granular Kidney and the Associated Cardio-arkrial Charges, 215
Joints, Various Wounds of Various, at Torbay Hospital (C.), 343
Juvenile Crime, 221
Kingsbury (Dr.) and Professional Advertising, 35
Kitson *versus* Playfair, 1
Lady Warwick's Home for Crippled Children, 164
Laminectomy in Potts' Disease and Spinal Injury (P.), 94
Lancaster New Infirmary (W.), 166
Landry's Paralysis (P.), 25
Laparotomy, Drainage after (P.), 263
Legalised Cruelty, 255
Leprosy (P.), 109, 303, 397
Leucocyte in Disease, The, 56
Leucocythemia (P.), 242
Leukæmia, Aural Complications of (P.), 245
Life and the Municipality, 253
Lightburn Joint Fever Hospital (W.), 250
Lister's (Sir Joseph) Address, 419
Lithiasis of the Gall Bladder (P.), 377
Lithotomy, Intraperitoneal Suprapubic (P.), 209
Liver, Diseases of the (P.), 409
Liver, Excision of a Portion of the, for Tumour (P.), 376
Liver, Hydatid Cyst of the (P.), 377
Local Authorities and Food Adulteration, 319
London University, 136
Long Bones, Rickety Deformities of the (P.), 282
Lumbar Puncture of the Sub-Arachnoid Space (P.), 93
Lunacy: Is Our Knowledge of Lunacy Stationary? 404
Lupus (P.), 308
Lymphadenoma (P.), 243
Lysol as an Antiseptic (P.), 412
Malaria (P.), 396
Malta Fever. *See p.* 207
Margarine and Deception, 405
Margarine, Science and Selfishness, 289
Marriage by Doctor's Consent, 85
Married, The Holidays of the, 303
Maunsell's Method of Intestinal Anastomosis (P.), 10
Maxillary Sinus (Cyst of) (P.), 111
Measles, The Municipal Control of, 152
Meat and Milk from Sewage Farms, 87
Meat, English and Foreign. *See p.* 247
Meatus, Exostosis of External (P.), 228
Meatus, External Auditory Suppositions, Foreign Bodies in (P.), 245
Meatus, Plugging of (P.), 228
Medical Aid Societies and Midland Doctors, 35
Medical Coercion, 387
Medical Schools, The Metropolitan, 372; Provincial, 373; Scotch, 375; Irish, 375; for Women, 375
Medical Student, The Career of the, 371
Medicine as a Profession, 368
Medicine as a Science or an Art, 386
Melbourne Homœopathic Hospital (W.), 364
Melksham Cottage Hospital (W.), 166
Meningitis, Basal. *See p.* 145
Mesenteric Cysts (P.), 360
Metropolitan Workhouse Children, 273
Microbe at Swansea, The, 387
Microbe, The, in Daily Life, 69
Microcephalus, Craniectomy for (P.), 143
Micro-organisms in the Inspired Air, What Becomes of? (P.), 127
Middle Ear Disease, Sequelæ of (P.), 160
Middle Ear, Tuberculous Disease of the (P.), 226
Middlesex Hospital (A.H.), 232
Midwives' Bills, 218
Midwives, Clipping the Wings of the, 86
Militant Charity, 5
Milk (P.), 25
Milk Dealers and the Law, 255
Milk for Infants, 387
Milk, Non-Saccharine for Diabetes (P.), 92
Modern Sociology, 105, 139, 155, 189, 205, 221, 257, 355, 370, 389, 407
Modern Sociology, The Foundations of, 173, 423
Moore, The late Surgeon-General Sir William James, K.I.C.E., 417
"Morbus Menierei" (P.), 227
Morphia Habit, The, 187
Mountain Air, The Influence of, 421
Murphy's Bullion (P.), 11, 360
Musculo-spiral Nerve, Suture of (P.), 77
Myocardium Affections of the (P.), 277
Nasal Affections, Associated with various Systematic Diseases, Note on (C.), 395
Nasal Obstruction (P.), 145
Nauheim Treatment, The (P.), 125, 142
Negro. Why is the Negro Black? 119
Nervous System, Diseases of the (P.), 24
Neuralgia, Trifacial, Operations for (P.), 75
Neuritis (P.), 24
New Appliances and Things Medical, 12, 26, 44, 60, 78, 96, 128, 146, 162, 179, 210, 228, 264, 296, 312, 328, 362, 398, 406, 428
New Northern Hospital, Liverpool (W.), 284
New Theology and the New Medicine, 89
Newcastle Hospital. Additions (W.), 365
Newton (Devon) Workhouse Infirmary (W.), 332
Night Shelters, and What we Pay for Them, 353
Night Terrors (Pavor Nocturnus), (P), 362
Northampton Infirmary (A.H.), 180
Notes and News, 6, 21, 36, 52, 70, 88, 104, 120, 138, 154, 172, 188, 204, 220, 238, 256, 274, 290, 304, 320, 338, 355, 389, 422
"Notification," The Ramifications of, 219
Nutrient Value (Comparative) of Meat Preparations, 115
Nystagmus (P.), 244
Obstetric Nurses this Time, 289
Obstetrics, Progress in, 178
Œdema, Subcutaneous Phlegmonous surrounding the Ear (P.), 244
Œsophagus and Stomach, Surgery of the (P.), 324
Ogston's Operation (Flat-foot), (C.), 7
Optic Neuritis in Brain Lesions (P.), 143
Orexin as a Gastric Stimulant, &c. (P.), 246
Ormskirk, New Cottage Hospital at (W.), 30
Osteomalacia (P.), 178
Otology (P.), 226, 243
Otorrhœa, Chronic. *See p.* 227
Otorrhœa, Stacke-Zaufel Operation (P.), 244
Oxygen in Surgical Cases (C), 222
Oxygen (Nascent), The Use of in Medicine (P.), 246
Oysters and Typhoid, Proposed Legislation, 118
Pancreas (P.), 191
Pancreas and Spleen, Surgery of the (P.), 26
Paralysis, Landry's (P.), 25
Parasitic Disease (P.), 309
Pelvic Exudations in Women, Treatment of (P.), 194
Pemphigus (P.), 308
Pensions for Asylum Officials, 20
Pericordial Effusion (P.), 260
Peritoneum, Structure and Absorption Power of the (P.), 263
Peritonitis (P.), 207
Peritonitis, General Septic (P.), 294
Pershore Cottage Hospital, 213, 267
Physic for Bad Temper, 255
Physic, The Young Ravens of, 317
Picric Acid in the Treatment of Burns (P.), 73, 107
Plague Spots of London, 219
Pneumonia (in children) (P.), 360
Pneumothorax, Clinical Lecture on (C.), 22, 38, 54
Population, Some Questions on, 5
Post-mortems on Hospital Patients, 87
Potts' Disease. *See page* 94
Practical Departments (W.), 31, 46, 212, 250, 267, 284, 349, 381
Practical Points (W.), 268, 315
Practice, Some Byeways of, 318
Pretoria, The Voice of Medical Science at, 135
Prevesical Inflammation (P.), 209
Prison Labour, 105, 139
Professional Advertising, 33 (*and see p.* 35), 301
Professional Secrecy and Ethics in General, 20
Prognosis, Sir D. Duckworth, 301
Prostate, Enlarged (P.), 177
Prostatectomy (P.), 178
Psoriasis (P.), 308
Purpura Hæmorrhagica following Malta or Mediterranean Fever, Case of (C.), 207
Pylorectomy (P.), 325
Pyloroplasty (P.), 325
Qualifying for Practice, 44
Quarantine in England, The Last of, 69
Rabbits—the Tit-bits of the Poor, 87
Radii, Congenital Absence of Both (P.), 280
Rate Supported Hospitals, 203
Rectal Exploration, Value of (C.), 376
Rectum, Cancer of the (P.), 356
Rectum, Carcinoma of the (P.), 311
Rectum, Stricture of the (P.), 312
Rectum, Villous Tumour of (P.), 312
Refuse, The Disposal of Town, 369
Renal Diseases (P.), 425
Renal Medicine (P.), 39
Respiratory Surgery (P.), 125
Retropharyngeal Abscess (P.), 362
Revaccination. *See p.* 103
Reynolds, The Late Sir Russell, 153
Rhinitis (P.), 126
Rhinitis, Fibrinous (P.), 127
Rheumatism, Gout and (P.), 158, 159
Rosario Hospital (W.), 330
Salicylic Acid, Therapeutic Action of (P.), 273
Salol, Local action of (P.), 43
Sanitary Officers and the Law, 68
Sanitation, Some Aspects of, 121
Scarlet Fever (P.), 108, 357
Science in Bonds: Quackery Free, 103
Scleroderma (P.), 309

Scraps and Gleanings, 14, 82, 133, 148, 166, 214, 232, 250, 268, 285, 315, 332, 349, 365, 382, 416
Sea Travel Without Sea Sickness, 239
Septicæmia (P.), 243
Serum Injection in Infectious Fevers (P.), 162
Sewage Farming and Sanitation, 201
Sewage Farms on Trial, 187
Sidmouth as a Health Resort, 51
Sigmoid Volvulus of the (P.), 12
Sinus (Sphenoidal), Abscess (P.), 95
Sinuses, Accessory, of the Nose (P.), 77
Sinusitis Antral (P.), 111
Sinusitis, Frontal and Ethmoidal (P.), 77
Small Hospitals, Special Departments at (W.), 13
Small-pox (P.), 108, 397
Small-pox at Gloucester, 35
Small-pox Infection. A Sanitary Shibboleth, 34
Small-pox, Joint Action Against, 117
Small-pox, The Early Diagnosis of, 340 (C.), 340
Sociology. *See Modern Sociology*
Special Departments of Small Hospitals (W.), 13
Spina bifida (P.), 94
Spinal Canal, Tumour of the (P.), 94
Spinal Cord, Tumours of the (C.), 157
Spine, Injuries to the (P.), 94
Spine, Lateral Curvature of the (P.), 280
Spleen (P.), 192
Spleen, Wandering, Splenopexis for (P.), 26
Splenectomy (P.), 27
Squint, Latent (C.), 156
State of the Streets, 254
Stokes's Operation (Flat Foot) (C.), 7
Stomach, Diseases of the (P.), 293
Stomach, Results of Operations upon the (P.), 326
Story of the Insane. *See Insane.*
Strake's Operation for the Cure of Chronic Otorrhea (P.), 227
Street Collections, 235
Street Collections (*note*), 19
Strophantus (P.), 278
Student of Medicine, The, 16, 31, 47, 66, 84, 100, 115, 134, 149, 168, 184, 200, 216, 234, 251, 269, 286, 300, 316, 334, 350, 366, 383, 402, 418, 433
Students and Maternity Departments, 351
Student to Practitioner, From, 367
Sub-Arachnoid Space, Lumbar Puncture of (P.), 93
Sub-diaphragmatic Abscess (P.), 125
Sub-maxillary Gland, Surgical Diseases of the (C.), 259
Subperiosteal Mastoid Abscess (P.), 161
Sugar, Proportion of, in Norman Urine (P.), 57
Suicidal and Dangerous Impulses in Insanity (C.), 90
Summer Suicides, 187
Suprarenal Capsules (P.), 192
Surgery of the Brain (P.), 109, 143, 160
Surgery, General, 41, 60, 411
Surgery, Genito-Urinary (P.), 177, 208, 225, 426
Surgery of the Intestines (P.), 9, 358
Surgery of the Liver and Gall Bladder (P.), 376
Surgery of the Nerves (P.), 75
Surgery of the Œsophagus and Stomach (P.), 326
Surgery of the Pancreas and Spleen (P.), 26
Surgery of the Peritoneum (P.), 294
Surgery of the Vermiform Appendix (P.), 345
Surgery, Respiratory (P.), 125
Sydney, Prince Alfred Hospital (A.H.), 432
Syphilis (P.), 226, 361
Syphilis, Treatment of, by Syphilitic Toxin (P.), 128
Tannigen in Diarrhœa (C.), 376
Tea (P.), 25
Temporal Bone, Localisation of Inflammations of the (P.), 245
Testis, Axial Rotation of the (P.), 225
Tetanus (P.), 59, 397
Theobromine (P.), 278
Therapeutic Notes (P.), 12, 43, 78, 112, 128, 146, 161, 210, 246, 295, 327, 346, 412
Thiersch's Skin Grafts (P.), 227
Thrombosis of Cerebral Sinuses (P.), 160
Thyroid Treatment in Sporadic Cretinism (P.) 376
Tobacco Pouches, Our, 405
Tongue, Cancer of the (C.), 306
Toronto General Hospital (W.), 430
Traumatic Cephalhydrocoele (P.), 144
Traumatic Epilepsy (P.), 144
Traumatic Insanity (P.), 145
Trunk, Postural Deformities of the (P.), 280
Tuberculosis, The Notification of, 217
Tuberculous Disease of the Bladder (P.), 426
Tuberculous Milk, Report of the Royal Commission, 49
Tuberculous Peritonitis, Laparotomy for (P.), 295
Tumour of the Spinal Canal (P.), 94
Tumours, Brain (P.), 109
Tumours of the Groin and their Differential Diagnosis (C.), 223
Tumours of the Spinal Cord (C.), 157
Tussol (P.), 346
Typhoid (P.), 58, 322
Typhoid and Oysters, 118
Typhoid Fever, Carbo-hydrate Starvation in, 3, *and see p.* 58
Typhoid. Is Typhoid a Preventable Disease? 69
Typhoid, Milk, in Scotland, 303
Typhoid Ulcers, Perforating (P.), 360
Ulcers, &c., Treatment by Oxygen (C.), 222
Ulcers, Perforating Typhoid (P.), 360
Ulcers, Varicose, of the Leg (C.), 424
University College Hospital (A.H.), 432
Unqualified Practice and the Law, 170
Upper Air Passages, Diseases of (P.), 77, 94, 111, 126, 145
Uræmia (P.), 425
Urethotomy, Perineal. *See p.* 225
Urethra, Rupture of (P.), 225
Urethra, Transplantation of, from Sheep (P.), 225
Urticaria (P.), 309
Uterus, Cancer of the (P.), 193
Vaccination. *See p.* 335
Vaccination, Insusceptibility to, 273
Vaccination Question, The, 115
Vaccination Report, The, 337, *and see p.* 335
Vaccination. Royal Commission Report. Full Analysis (C.), 390
Vaccination, The Future of, 101
Vaccination, The Medical Attitude Towards Compulsory, 352
Vaccination, The Report of the Royal Commission on, 385
Vaginal Cœliotomy (P.), 194
Varicose Ulcers of the Leg (C.), 424
Vasa Deferentia, Ligation of (P.), 426
Ventilation, Mechanical, of Hospital Wards (W.), 249, 268
Ventilation Plenum and Hospital Plans (W.), 400
Vermiform Appendix, Surgery of the (P.), 345
Vinegars, About, 319
Volunteer Medical Staff Corps at Walmer (W.), 45
Volvulus of the Sigmoid (P.), 12
Waddon Isolation Hospital (W.), 332
Walsall Workhouse Infirmary (W.), 165
Wandering Spleen (P.), 26
Water Supply of Small Towns, The, 50
Wells, New Cottage Hospital at (W.), 231
Why Hospital Patients Complain (W.), 64
Worsted, The Surgical Uses of, 421

### THE SPECIAL SUPPLEMENT FOR NURSES.

Actor's Orphanage Fund, The, cxxiv

Adelaide Hospital, The, xxxiv

American Letter, Our, xxv, lxxxii, ciii, cxxxiii

Appointments, vi, xix, xxvi, xxxv, xlii, l, lx, lxiv, xciv, cii, cxii, cxx, cxxviii, cxlvi, cliv, clxv, clxxiii, clxxx, cxciv, ccv, ccxviii, ccxxvi

Appointments, Minor, iv, xx, xli, l, lvi, lxvi, lxxii, xc, cii, cxxviii, cxxxv, cxlii, clvi, clxvi, clxxiii, clxxx, clxxxix, cxcvi, ccvii, ccxvii, ccxxvii

Asylum News and Nursing, clv

Bolingbroke Hospital, Wandsworth Common, lxxxiii

Book and its Story, A, ix, xviii, xxvii, lvii, lxxvii, xciii, cxix, clxiii, clxxii, cxcvii, ccxvi

Book World for Women and Nurses, x, lx, cxxxvi, clxxiv, cc, ccxxiv

City of London Union Infirmary, clxxix

Cottage Nurses, cix

Death in our Ranks, xv, xlii, xlix, cxi, clxxiii, clxxxix, ccv

Difficulties of a Provincial Matron, xiv

District Nurses and Infectious Diseases, cxcviii

District Nursing in Holland, clxx

Dressmaking Classes. clxxxvi

Dress and Uniforms, xvii, lxxxv, cxxxiv, clxxxi, ccxiv

Everybody's Opinion, viii, xix, xxviii, xxxv, xlii, l, lix, lxvii, lxxxvi, xcv, ciii, cxi, cxx, cxxxv, cxlv, clv, clxvi, clxxiii, clxxxix, cxcix, ccvii, ccxxvi

Experience in the London Hospital, An, xciv

Generous Help, xxvi

Holidays and Health, lxxv, cii, cxviii, clxiii, clxxx, clxxxviii

Holiday on Robben Island, A, cxcvi

Home Hospital, The First and Best, xxxiii

Hospital Life, Sketches of, civ

How Small-pox is Nursed in Gloucester, lv, lxxiv

Hygiene for Nurses, xiii, xxiii, xxxi, xxxix, xlv, liii, lxiii, lxxi, lxxxi, lxxxix, xcix, cvii, cxv, cxxiii, cxxxi, cxli, cxlix, clix, clxix, clxxvii, clxxxv, cxciii, cciii, ccxi, ccxxi

Indian Nursing Service, The, ci, clxxxvii, *and see p.* xlviii

Jottings from the Punjab, ccxxiv

Junius S. Morgan Benevolent Fund, The, cl

Lady Dufferin's Fund, cxxiv

Lying-in Hospitals and Treatment by Midwives, lxxiv

Male Nurses, The Training of, xliv

Mary Wardell Convalescent Home, The, clxv

Maternity Charity and District Nurses' Home, cx

Matron, The (poem), cxcviii

Medals at the Cookery Exhibition, vii

Medical Missions in India, cxciv

Middlesex Hospital, Grand Fête at the, cxxiv

Midwifery Papers, v, xcii, cviii, ccxxii

Mission to Deep Sea Fishermen lxvi

National Hospital for the Paralysed and Epileptic, Queen's Square, lvi

News from India, South Travancore, cxvii

News from the Nursing World, i, xi, xxi, xxix, xxxvii, xliii, li, lxi, lxix, lxxix, lxxxvii, xcvii, cv, cxiii, cxxi, cxxix, cxxxvii, cxlvii, clvii, clxvii, clxxv, clxxxiii, cxci, cci, ccix, ccxix

New York Training School for Nurses, cl

Nightingale Home, St. Thomas' Hospital, The, cx

Nightingale Nurses, cliv

North-Eastern Hospital for Children, The, cxii

Notes from Melbourne. cx

Notes and Queries, viii, xx, xxvi, xxxvi, xlii, l, lx, lxviii, lxxviii, lxxxvi, xcvi, ciii, cxii, cxx, cxxviii, cxxxvi, cxlvi, clvi, clxxiii, clxxxii, cxc, cc, ccviii, ccxviii, ccxxvi

Novelties for Nurses, vii, xix, xlii, xlvii, lviii, lxxvi, cxxviii, cliv, clxii, cciv, ccxvii

Nurse, A Brave, xciii

Nurse Gossip, The, xxvi

Nurse, The Visiting, lxxxiv

Nurses' Association, Royal British, xxiv, cxxxv, cxliv, clii, clxii, clxxxii

Nurses' Clinic, Trained, xv, xxv, xl, liv, lxvi, lxxii, c, cxvi, cxxxii, clx, clxxxvi, cxciv

Nurses' Co-operation, The, v

Nurses, Cottage, cix

Nurses in 1896—Their Quarters, Hours, and Food, cli, clxi, clxxi, cxcv, ccv, ccxv, ccxxv

Nurses' Pensions, Sir Benjamin Richardson on, lv

Nurses, The Society of Chartered, vii

Nurses' Visit to Texas, A, ccxvii

Nursing in Alexandria, ccxxiii

Nursing Appliances, Exhibition of, lxxxiv

Nursing Arrangements at Clapham, cxlv

Nursing Association, The Colonial, cxxxiii, cxlii

Nursing Association, Metropolitan, xlix

Nursing in Buenos Ayres, lviii

Nursing Conference at St. Martin's Town Hall, xci

Nursing in Crown Colonies, vii

Nursing, Ethics of, clii, clxv, clxx, clxxviii

Nursing Exhibitions, xcvi

Nursing Question as seen in England and Germany, Some Aspects of the, iii, xvi, xxxiv, lxxiii, lxxxiii, cliii

Poor Law Infirmary Nurses and the Superannuation Bill, xli

Practical Points, viii

Presentations, xix, xxvii, xlix, lx, lxvi, cxxxv clii

Prince and Princess Charles of Denmark, T.R., cxxxix

Private Case in the Country, A, iv

Queen Victoria's Jubilee Institute for Nurses, cxlvi

Queen's Nurses, New, cxxvi

Queen's Nurses, Parade of, clii

"Queen's Nurses," Reception of, by Her Majesty at Windsor, cxxv

Reading to the Sick, For, x, xx, xxxvi, lviii, lxviii, xcvi, civ, cxxviii, cxlvi, clxvi, clxxxii, cxc, ccviii

Royal Wedding, The, cxl

St. Olave's Guardians and the Matron of the Infirmary, The, cxxvii

St. Olave's Union Infirmary, cxxxiii

Smallpox. How Smallpox is Nursed at Gloucester, lv, lxxiv

South Africa and its Institutions, ccxiii

South African Asylum, Some Aspects of Life in a, lxv, xc

South African Hospital, A, cxlii

Summer Exhibitions, The, lvi

Talks about Food, vi

"Truth" and the Indian Nursing Service, xlviii

"The Power for the Children," cxvii

Unemployment of Atkins, The, cxliv

Wants and Workers, viii, xx, xxiv, xxxiii, xl, l, lx, lxxiii, xc, civ, cix, cxx, cxxxii, cxlvi, cciv

Where to Go, xix, xxiv, xxxv, xlii, lvi, lxiv, lxxviii, lxxxii, xciv, ci, cxii, cxviii, cxxxii, cxlvi, cciv

Women as Sanitary Inspectors, cxi

Workhouse Infirmary Nursing Association, xxxii, lxiv

Workhouse Infirmaries and their Nurses, ccvi, ccxii

Workhouse Reform, Irish, xlvii

Zenana Bible and Medical Mission, xcv

Printed by W. H. and L. Collingridge, "City Press," 148 and 149, Aldersgate Street, London, E.C.

**Notices of Births, Deaths, and Marriages** are inserted in this column at a charge of 2s. 6d. for an announcement not exceeding four lines.

**Notice to Advertisers and Subscribers.**

All Advertisements, Orders for Copies of The Hospital and Business Communications should be addressed to **THE MANAGER, (Not to The Editor)**, The Hospital, 428, Strand, London, W.C.

The Cost of Subscription, post free, is as follows:—Per week, 2½d.; per quarter, 2s. 8d.; per half-year, 5s. 3d.; per annum, 10s. 6d. Foreign:—Per Annum, 15s. 2d.; payable, in all cases, in advance.

### NOTICE TO CORRESPONDENTS.

All MS., Letters, Books for Review, and other matters intended for the Editor should be addressed The Editor, The Lodge, Porchester Square, London, W.

The Editor cannot undertake to return rejected MS., even when accompanied by stamped directed envelope.

Ready Shortly.

**"Burdett's Hospitals and Charities, 1896."**

Seventh year of publication. Over 1,000 pp. Scarlet cloth, gilt lettered. Price 5s. A most complete guide to British, Colonial, Indian, and American Hospitals, Dispensaries, Nursing and Convalescent Institutions, and Asylums. A few complete sets of the preceding six issues of the "Annual" may still be obtained on application to the Publishers, The Scientific Press (Limited), 428, Strand, London, W.C.

**NOTICE.—Vol. XIX. of "The Hospital" (October, 1895, to March, 1896), in handsome cloth binding, will be on sale at the Office of this Journal early in April. Price 7s. 6d. Cases for binding the Numbers contained in this Volume, 1s. 6d. Index to Vol. XIX., 2½d., post free.**

**The Monthly Part for April is now ready, price 6d., by post, 8d.**

### Notes and News.

### THE METROPOLITAN HOSPITAL SATURDAY FUND.

### SCRAPS AND GLEANINGS.

The warehouse of the Rosbach Springs (Limited) is removed from 4, Sussex Place, E.C., to 20, Bury Street, E.C. All communications should be addressed to the offices of the company, 38, Leadenhall Street.

At the conclusion of the recent annual court of governors of the Royal Free Hospital the marble memorial of the late Mr. George Moore was unveiled in the Moore Ward. The memorial is in recognition of the valuable services rendered to the hospital by Mr. Moore, who was chairman for six years.

The Sussex Eye Hospital has received a cheque for £200 from Miss L. da Costa, making a total sum of £1,300 given by this lady at various times to the institution.

It was stated at the annual meeting of the governors last month that the economical management of the Bradford Children's Hospital compares very favourably with other children's hospitals. There is, however, a balance still due on the building account of about £1,600, and an accumulation of annual deficiencies amounting to £806 on the general account.

The record of the Rochdale Infirmary and Dispensary during the year 1895 is, on the whole, fairly satisfactory from a financial point of view, though there is yet much room for improvement.

### THE STUDENT OF MEDICINE.

### APPOINTMENTS.

R. Brayn, L.R.C.P.Lond., M.R.C.S.Eng., appointed Superintendent to the State Criminal Lunatic Asylum, Broadmoor. W. C. Brown, B.A.Lond., M.B., Ch.B.Vict., appointed Junior Assistant Resident Surgeon to the New Somerset Hospital, Cape Town. P. G. Cilliers, M.B., C.M., appointed Clinical Assistant to Chelsea Hospital for Women. G. Findlay, M.A.Aberd., M.B., C.M., appointed Medical Officer of Health for the Campden Urban District. C. W. Graham, L.F.P.S.Glasg., L.R.C.P., appointed Honorary Medical Officer to the Carlisle Dispensary. C. B. Hall, M.R.C.S., L.R.C.P., appointed Resident Medical Officer to the Chelsea Hospital for Women. S. C. Hounsfield, M.R.C.S.Eng., L.R.C.P.Lond., appointed second House Surgeon to the East Suffolk Hospital, Ipswich. A. Jefferson, M.D. and B.S.Lond., M.R.C.S.Eng., appointed Honorary Medical Officer to the Rochdale Infirmary and Dispensary. S. A. Jolly, appointed Medical Officer to the Goldsmiths' Almshouses, Acton. R. T. Jones, L.R.C.P., L.R.C.S.Edin., L.F.P.S.Glag., appointed Deputy Medical Officer for the No. 1 District of the Llandovery Union. W. B. Jones, M.D., B.S.Lond., appointed Resident Physician at the Bathing Establishment at Llangammarch Wells, Central Wales. W. J. Kerr, M.D., Ch.B.Vict., M.R.C.S.Eng., L.R.C.P.Lond., appointed Honorary Surgeon to the Rochdale Infirmary. C. Lamplough, M.R.C.S.Eng., L.R.C.P.Lond., appointed House Physician, City of London Hospital for Diseases of the Chest, Victoria Park. Dr. Mackenzie, appointed Medical Officer for the Fifteenth District of the Basford Union. W. W. Robinson, L.R.C.P., L.R.C.S.Edin., appointed Medical Officer for the Parish of Hasland of the Chesterfield Union. J. R. Russell, M.D.Edin., M.R.C.P., appointed Pathologist to the National Hospital for the Paralysed and Epileptic, Queen Square. A. E. Sellers, L.R.C.P.Lond., M.R.C.S., appointed Medical Officer of Health for the Thornhill Urban District. Dr. J. H. Stewart appointed Medical Officer of Health for the Borough of Lostwithiel. M. Townsend, L.S.A., appointed Second Assistant Medical Officer to the Marylebone Infirmary. G. E. Walker, L.R.C.P.Lond., M.R.C.S.Eng., appointed Governor and Medical Officer to the Aylesbury Female Convict Prison.

### VACANCIES.

The following vacancies are announced this week, the amount of salary in each case being given after the name of the institution:—

### Resident Medical Officers.

City of Birmingham.—Deputy Medical Superintendent for the City Hospital, Little Bromwich, under 40 years of age, unmarried, and doubly qualified. Salary £175 per annum, with residence, rations, and attendance. Applications, endorsed "Deputy Medical Superintendent," to be sent to Mr. J. Keyte, Clerk to the Health Committee, Council House, Birmingham, by April 14th.

Donegal District Lunatic Asylum, Letterkenny.—Assistant Medical Officer; qualified in medicine, surgery, and midwifery; unmarried, and not more than 30 years old. Salary £100 per annum, with furnished apartments, rations, washing fuel, light and attendance, valued at £100 per annum. Applications to Dr. Moore, Resident Medical Superintendent, by May 9th.

Dundee Royal Lunatic Asylum.—Assistant Medical Officer. Salary £100 per annum, with board, lodging, &c. Applications to Dr. Rorie at the Asylum by April 4th.

East London Hospital for Children, Glamis Road, Shadwell, E.—House Surgeon. Board, lodging, &c., provided, but no salary. Applications to the Secretary by April 4th.

Hereford County and City Asylum.—Medical Superintendent. Salary £400 per annum, with furnished house, coals, gas, vegetables, and washing. Applications to the Chairman, Asylum Committee, Shire-hall, Hereford, by April 28th.

Hospital for Diseases of the Chest, City Road, E.C.—Resident Medical Officer. Appointment for six months, but eligible for re-election. Salary at the rate of £100 per annum, with furnished apartments, board, and washing. Applications to the Secretary at the Hospital by April 13th.

Kesteven and Grantham District Asylum.—Medical Superintendent. Salary £300 a year, with rations, coal, light, and washing. Must devote his whole time to the duties of the office. Applications to Jos. Phillips, Clerk to the Visitors, Stamford, by April 11th.

Miller Hospital and Royal Kent Dispensary, Greenwich Road, S.E.—Junior Resident Medical Officer. Appointment for six months, with prospect of re-election. Salary £30 per annum, with board, attendance, and washing. Applications to the Honorary Secretary or Secretary by April 11th.

Paddington Green Children's Hospital, W.—House Physician and House Surgeon. Appointments for six months. Salary at the rate of £50 per annum, with board and residence. Applications to the Secretary by April 10th.

Parish of Birmingham Workhouse Infirmary.—Resident Assistant Medical Officer of the Workhouse Infirmary. Salary £100 per annum, with furnished apartments, rations (which do not include alcoholic liquors), coals, gas, washing, and attendance. Application on forms provided to be sent to Walter Bowen, Clerk to the Guardians, Parish Offices, Edmund Street, Birmingham, by April 4th.

### Non-Resident Medical Officers.

Chelsea Hospital for Women, Fulham Road, S.W.—Surgeon. Applications to the Secretary by April 4th.

Chichester Infirmary.—Honorary Medical Officer. Applications to the Secretary by April 9th.

City of London Hospital for Diseases of the Chest, Victoria Park, E.—Assistant Physician; must be F. or M.R.C.P.Lond. Applications to the Secretary by April 9th.

New Hospital for Women. 144, Euston Road, N.W.—Qualified Medical Woman as a Clinical Assistant in the Out-patient Department, and also Assistant Anæsthetist. Appointments for one year. Applications to the Secretary by April 8th.

Royal College of Physicians.—Milroy Lecturer. Applications to the Registrar, Royal College of Physicians, Pall Mall East, by April 9th.

St. Mary's Hospital Medical School, Paddington.—Bacteriologist. Applications to the Dean by April 11th.

